# A Textbook of General Bacteriology

**Published:** 1910-12

**Authors:** 


**A Textbook of General Bacteriology.** By **Edwin O. Jordan,** Ph.D., Professor of Bacteriology in the University of Chicago and in Rush Medical College. Second edition. Octavo of 557 pages. Illustrated. Philadelphia and London: W. B. Saunders Co. 1910. (Coth, $3.00 net.)

The medical aim of the future is prevention, and bacteriology is her handmaid. The science of bacteriology is growing at a rate beyond precedent; it cannot grow too fast for the needs of preventive medicine.

This work is a scientific contribution of distinct and unusual value, comprehensive and satisfactory in scope and treatment. We are particularly pleased with the author's intellectual honesty and courage. He frequently remarks, "we do not know;" "it is not known;" "we may never know." This shows that the author is worthy of confidence, because he has that priceless gift, the poise of true scholarship. The author also is pleasing in his clarity of style. His paragraphs frequently open with a concise statement and close with an important and definite conclusion. For instance, in chapter VII, Immunity, we read:

"Immunity, like pathogenicity, is a relative term; all living organisms, at least all the higher forms, are susceptible under some conditions to some kind of parasitic invasion."

"On the other hand, some degree of resistance against parasitic attack seems to be manifested by all animals and plants."

"The wild carnivora, for example, are probably exempt from bacterial infections."

"Man is susceptible to infection with a great variety of micro-organisms, certain of which possess little or no pathogenic power for any other animals."

"Little is clearly known about the chemical character of the antitoxins."

"Such facts indicate that the antitoxin is probably generated in the tissues."

"So far as has been discovered the antitoxic substances present in the body of the normal organism are identical with those found in actively immunised animals."

"Every physiologic consideration points to the body cells as the place of origin of the antitoxins."

"Animal blood cannot only neutralise bacterial toxins, but can destroy bacteria themselves."

"The diapedesis of the white corpuscles, their migration through the vessel walls into the cavities and tissues, is one of the principal means of defense possessed by an animal."

The table of contents of thirty-five chapters and an appendix, with abundant and accurate subheads, a copious bibliography, a minute and ample index, help to make this book one which busy medical men will appreciate and use, and from thorough acquaintance will come to love and cherish.

